# Supplementary material for: Evaluating the impact of an intervention to increase uptake of modern contraceptives among adolescent girls (15–19 years) in Nigeria, Ethiopia and Tanzania: the Adolescents 360 quasi-experimental study protocol
Source: BMJ Open. 2018 May 31;8(5):e021834. doi: 10.1136/bmjopen-2018-021834 (PMC5988138; doi:10.1136/bmjopen-2018-021834)
Supplement: Supplementary file 1 [file bmjopen-2018-021834supp001.pdf]

A360 protocol- Further details on sample size calculations

Table S1: Baseline estimates of mCPR

Setting	Population	mCPR among 15-19 year olds in the most recent DHS available when planning study[1]	PSI estimated mCPR in 2015[2]	PSI projections of annual temporal trend[2]	PSI estimated mCPR in 2017[2]	Study protocol estimated mCPR in 2017[3]
Ethiopia	Married 15-19 year olds	39.6%	39.6%	2.2%	44.0%	44.0%
Nigeria	Married 15-19 year olds	1.2%	1.2%	0.3%	1.8%	3.0%
	Unmarried Sexually active 15-19 year olds	49.7%	53.0%	2.0%	57.0%	64.4%
Tanzania	Married 15-19 year olds	12.0%	15.0%	0.6%	16.2%	16.2%
	Unmarried Sexually active 15-19 year olds	34.5%	37.5%	0.5%	38.5%	38.5%

[1]Ethiopia mini DHS 2014; Nigeria DHS 2013; Tanzania DHS 2010

[2]PSI projections of mCPR in the absence of intervention (source: PSI internal document 'A360 New User Estimates_24 Feb 2016.xls').

[3]mCPR baseline estimates for this study were in line with PSI estimates except for in Nigeria where we estimated a higher baseline mCPR as the mCPR among married women 15-49 years in our study states Nasarawa (16.3%) and Ogun (21.5%) were higher than the national mCPR (9.8%) (DHS 2013, Table 7.4).

Table S2: Estimates of impact

		mCPR in 2017[1]		Temporal trend (per year)[2]	Intervention impact (per year)[3]	mCPR in 2019[1]		Minimum measurable effect (% increase over 2 years in A360 areas)	% point increase over 2 years in A360 areas
	**Population**	**non-A360 areas**	**A360 areas**			**Non-A360 areas**	**A360 areas**		
**Ethiopia**	**Married**	44.0%	44.0%	1.5%	1.9%	47.0%	50.8%	15%	6.8%
**Nigeria**	**Married**	3.0%	3.0%	0.05%	1.0%	3.1%	5.1%	70%	2.1%, DID 2.0%
	**Unmarried Sexually active**	64.4%	64.4%	0.6%	3.5%	65.6%	72.6%	13%	8.2%, DID 7.0%
**Tanzania[4]**	**Married**	16.2%	16.2%	0.94%	1.8%	18.1%	21.7%	34%	5.5%
	**Unmarried Sexually active**	38.5%	38.5%	1.22%	2.0%	40.9%	44.9%	17%	6.4%
	**Total sexually active[3]**	26.7%	26.7%	1.1%	1.9%	28.9%	32.7%	22%	5.9%

[1] Based on PSI projections (Table S1)

[2] The estimated annual increase in mCPR in the absence of A360 (temporal trend) was estimated using historical DHS and PMA2020 data

[3] Projected study effect sizes were informed by a review of previous evaluations of interventions to increase the use of modern contraceptives that was conducted by Michelle Weinberger. Her review revealed that the median OR in reviewed studies was 1.36 and that the annual increases in mCPR from pre-post studies ranged from 1.3% to 8.5%. We estimated that A360 would result in a median ~1-2% annual increase in mCPR for married girls and a median ~2% annual increase in mCPR for unmarried girls. Among unmarried girls in Nigeria (Ogun State), we predicted a 3.5% annual increase in mCPR.

[4] In Tanzania, estimates and assumptions were made for married and unmarried 15-19 year olds separately, then estimates were combined assuming 21.7% of 15-19 year olds were married and 19.4% of unmarried girls were sexually active.

Table S3 Design Effect

Setting	***ICC[1]***	***Number of clusters***	***Eligible girls/cluster***	***Design effect (DE)[2]***
Nigeria – Nasarawa	*0.04*	*1148*	*4*	*1.12*
Nigeria- Ogun	*0.01*	*708*	*17*	*1.16*
Ethiopia	*0.02*	*45*	*28*	*1.54*
Tanzania- scenario 1	*0.005*	*30*	*110*	*1.5*
Tanzania- scenario 2	*0.012*	*30*	*84*	*2.0*

[1]The Intracluster correlation coefficient (ICC) is the ratio of the between-cluster variance to the total variance (both between and within clusters), and has a value between 0 and 1. If ICC is 0 then there is no clustering so individuals within clusters are no more similar than individuals from different clusters. ICC estimates for this study were based on ICC reported in the literature and our knowledge of the prevalence of the outcome, size of cluster, likelihood of diversity within clusters.
(Pagel et al, Trials 2011 (https://trialsjournal.biomedcentral.com/articles/10.1186/1745-6215-12-151)).
[2] DE = (1+ICC (cluster size-1))

Table S4 Sample size for adults

Study design	***Outcome 2017***	***Outcome 2019***	***Number needed (n)***	***DE***	***Total sample of adults (n X DE)***	***Protocol target sample size***
Difference in differences	*Intervention 40%*<br>*Comparison 40%*	*Intervention 45%*<br>*Comparison 63%*	*232*	*1.1*	*255*	*250*
Before-after study design	*40%*	*62%*	*80*	*1.5*	*120*	*127/128*

Note: We calculated the target number of adults to have 80% power to detect an increase in an attitudinal variable (undefined) from approximately 40% in 2017 to approximately 60% in 2019. The final sample size of adults was then adjusted slightly to be a fixed proportion of the target number of girls to be interviewed.
